# Improved synthesis of antiplasmodial 4-aminoacridines and 4,9-diaminoacridines†

**DOI:** 10.1039/d4ra00091a

**Published:** 2024-02-19

**Authors:** Mélanie Fonte, Cátia Teixeira, Paula Gomes

**Affiliations:** a LAQV-REQUIMTE, Departamento de Química e Bioquímica, Faculdade de Ciências Universidade do Porto Portugal pgomes@fc.up.pt

## Abstract

Acridines are one of the most important nitrogen-containing heterocycle systems and have many applications in the therapeutic field. However, the synthesis of acridine-based scaffolds is not always straightforward. Herein, we report the optimization of two multi-step synthetic routes towards 4,9-diaminoacridines and 4-aminoacridines, which have shown promising antiplasmodial properties. The improved synthesis pathways make use of greener, simpler, and more efficient methods, with less reaction steps and increased overall yields, which were doubled in some cases. These are impactful results towards future approaches to the chemical synthesis of acridine-based compounds.

## Abstract 2

Greener, simpler and higher yield methods are greatly desirable for the production of acridines, given their relevance in the therapeutic field. Herein, we report an improved multi-step synthesis of antiplasmodial acridines.

## Graphical abstract

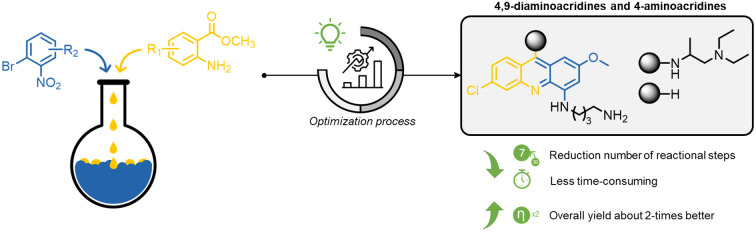

## Introduction

**Fig. 1:**
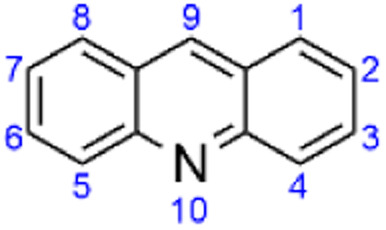
Acridine structure and its numbering as defined by the International Union of Pure and Applied Chemistry (IUPAC).

Acridine (Fig. 1) was initially extracted by Grabe and Caro from the anthracene fraction of coal tar in 1870.^1,2^ Due to its unique physical and chemical properties, biological activities, and industrial applications, it represents one of the most important nitrogen-containing heterocycle systems. Acridines were first used as dyes in fabrics, but their fluorescence properties soon after triggered their wide application in several cellular investigations such as cell cycle determination, nucleic acid staining, and flow cytometry. The therapeutic potential of aminoacridines, owing to their antimicrobial properties, was recognized as soon as in 1912 and underpinned the use of acridines as antiseptics during World War I. The therapeutic relevance of acridine-based compounds was further consolidated in World War II, when mepacrine, a 9-aminoacridine, was widely employed as an antimalarial drug to compensate for the ongoing chloroquine shortage. Since then, both mepacrine and other acridine derivatives have been reported with antitumoral, antibacterial, anthelmintic, antiviral, antifungal, antitubercular, and antileishmanial activities, among other potential therapeutic uses, including for neurodegenerative diseases.^1–6^

There is no general method for the synthesis of acridine derivatives. Direct functionalization of the acridine ring is a most interesting method, since it reduces the number of synthetic steps required, facilitating the synthesis of complex polycyclic structures. However, these approaches have a limited range of applications, as classic electrophilic substitutions on the acridine ring are usually not regioselective and produce many polyfunctionalized products. In this sense, the most widely applied syntheses of acridines involve, as a first step, ring closure through the condensation of adequately functionalized anilines and *o*-halobenzoic acids. The Bernthsen reaction was one of the earliest methods to obtain the acridine core and consists in mixing an aromatic or aliphatic carboxylic acid with a diphenylamine in the presence of zinc chloride and heating at high temperature (200–270 °C) for several hours.^7^ Due to these harsh conditions and to the limited variety of substrates available, this method was quickly superseded by copper- or palladium-catalyzed methods, namely, Ulmann and Buchwald–Hartwig cross-coupling reactions, respectively, followed by dehydrogenation.^1,6,8,9^ The acridine core obtained *via* the aforementioned methods is a versatile scaffold that can undergo many further modifications, mainly in the C-9 and N-10 positions, which are the most reactive sites due to the presence of the pyridinic nitrogen in the heterocycle. The reactivity of these positions is obviously modulated by the electron-donating or withdrawing properties of the substituents on the phenyl rings of the acridine scaffold.^6,10,11^ The N-10 position is the main nucleophilic site of the acridine core, in which *N*-alkylations can easily occur, with the formation of acridinium salts under strongly acidic conditions. On the other hand, due to the strong electron deficiency caused by the pyridinic nitrogen on the C-9 position, this is the most electrophilic site of the acridine system, allowing for nucleophilic reactions to take place there.^10^ For these reasons, most of the acridine-based compounds developed so far display chemical modifications in those two positions,^12,13^ while modifications elsewhere in the acridine ring remain limited.^13^

**Scheme 1:**
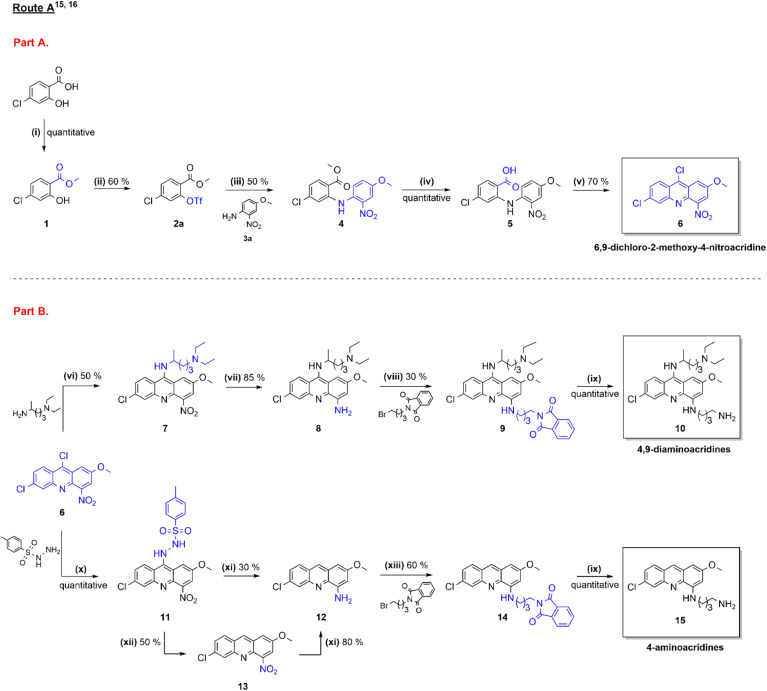
Synthetic route A towards 6,9-dichloro-2-methoxy-4-nitroacridine (6), 4,9-diaminoacridines (10) and 4-aminoacridines (15), as previously reported by Fonte M. *et al.*^15,16^ Reagents and conditions: (i) CH_3_I (5 molar equivalents, equiv.), Cs_2_CO_3_ (0.5 equiv.), dimethylformamide (DMF), room temperature (rt), 1.5 h; (ii) Tf_2_O (1.5 equiv.), Et_3_N (2 equiv.), CH_2_Cl_2_, N_2_ atmosphere, −25 °C, 30 min; (iii) 4-methoxy-2-nitroaniline (3a, 1.2 equiv.), Cs_2_CO_3_ (1.4 equiv.), Pd(OAc)_2_ (0.05 equiv.), *rac*-BINAP (0.08 equiv.), toluene, N_2_ atmosphere, 120 °C, 5 h; (iv) (1) Ba(OH)_2_·8H_2_O (1.5 equiv.), CH_3_OH, 90 °C, 2 h; (2) 1 M aq. HCl; (v) POCl_3_ (34 equiv.), 120 °C, 2.5 h; (vi) (1) phenol (15 equiv.), Cs_2_CO_3_ (1 equiv.), anhydrous dimethyl sulfoxide (DMSO), 4 Å molecular sieves, 100 °C, 2 h; (2) *N*′,*N*′-diethylpentan-1,4-diamine (4 equiv.), 100 °C, 4 h; (vii) SnCl_2_ (5 equiv.), 37% aq. HCl, 0 → 40 °C, 30 min; (viii) *N*-(4-bromobutyl)phthalimide (3 equiv.), CH_3_COONa (3 equiv.), CH_3_CH_2_OH, microwave (MW) heating (100 W, 120 °C) in a pressurized vial (100 psi), 2.5 h; (ix) hydrazine monohydrate (40 equiv.), tetrahydrofuran (THF), 80 °C, 24–48 h; (x) *p*-toluenesulfonyl hydrazide (*p*-TSH, 1 equiv.), CHCl_3_, rt, 24–48 h; (xi) NH_2_NH_2_ (10 equiv.), Pd/C (10% wt), CH_3_OH, 80 °C, 1–3 h; (xii) HOCH_2_CH_2_OH/H_2_O (2 : 1), Na_2_CO_3_ (0.0625 M), 95 °C, 1.5 h; (xiii) *N*-(4-bromobutyl)phthalimide (3 equiv.), Et_3_N (3 equiv.), CH_3_CH_2_OH, MW heating (100 W, 120 °C) in a pressurized vial (100 psi), 3 h.

Based on the well-known therapeutic potential of aminoacridines,^14–17^ our research group recently developed two unprecedented synthesis routes towards 4,9-diaminoacridines and 4-aminoacridines (Scheme 1).^15,16^ These routes comprise two main parts: (a) production of the 6,9-dichloro-2-methoxy-4-nitroacridine precursor common to both 4,9-diaminoacridine and 4-aminoacridine target families (Scheme 1, part A), and (b) further modifications in C-9 and/or C-4 positions of the acridine ring (Scheme 1, part B).^15,16^ Both are extensive and laborious synthetic routes that involve several steps featuring harsh reactional conditions and low yields. Based on this, and further motivated by the fact that these families of aminoacridines have shown interesting antiplasmodial properties,^16^ we have now investigated whether simpler, more selective, and less time-consuming methods could be used to synthesize the same target aminoacridines in fewer reactional steps and with improved yields. Successful improvement of the synthesis routes was achieved, as herein reported.

## Results and discussion

### Optimization of the synthesis route to 6,9-dichloro-2-methoxy-4-nitroacridine (6)

6,9-Dichloro-2-methoxy-4-nitroacridine (6) is the main synthetic precursor of acridines modified in the C-4 and C-9 positions. We have previously achieved the synthesis of 6, starting from commercial 4-chlorosalicylic acid that was first protected by conversion into its methyl ester (1, Scheme 1, step i), then activated by conversion of the alcohol into a triflate (2a, Scheme 1, step ii), which was next reacted with 4-methoxy-2-nitroaniline (3a) *via* the Buchwald–Hartwig coupling reaction (Scheme 1, step iii). The product obtained (4) was next submitted to basic hydrolysis (Scheme 1, step iv) to afford the carboxylic acid 5, and this was then treated POCl_3_ (Scheme 1, step v) to promote the desired cyclization into 6,9-dichloro-2-methoxy-4-nitroacridine 6 (Scheme 1, part A).^15^ This synthetic route was adapted from the literature,^18^ and was the only one that, in our hands, successfully led to the target precursor 6, which we recurrently failed to produce by means of Ulmann-type reactions.^15^ Still, this synthetic route to 6 posed a few challenges, mainly due to the strong electron-withdrawing effect of the nitro group that significantly decreased the reactivity of the aniline 3a in the Buchwald–Hartwig coupling. While this was compensated by use of an excellent leaving group such as triflate, triflate 2a is not commercially available, which added three more steps to the synthesis route (Scheme 1, steps i, ii and iv).

**Scheme 2:**
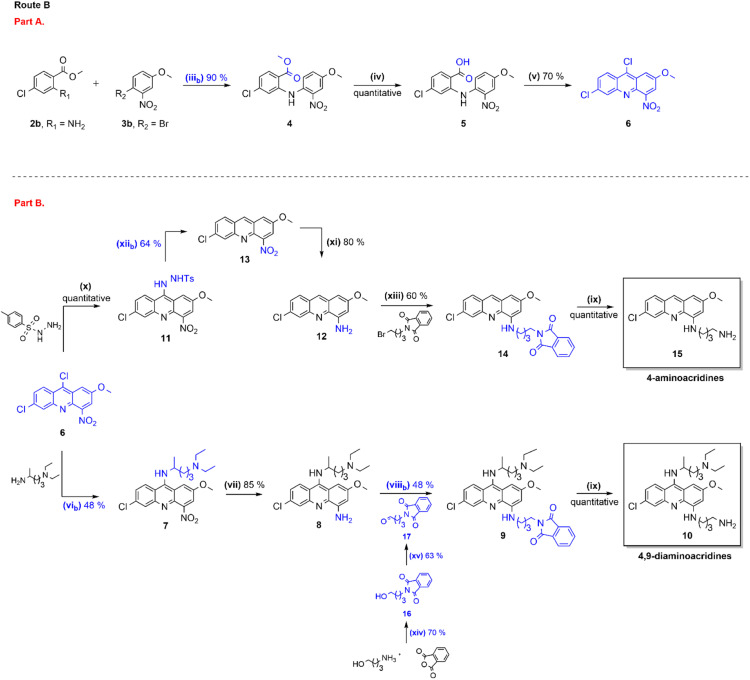
Synthetic route B towards 6,9-dichloro-2-methoxy-4-nitroacridine (6), 4,9-diaminoacridines (10) and 4-aminoacridines (15). Reagents and conditions: (iii_b_) 2b (1 equiv.), 3b (1 equiv.), Cs_2_CO_3_ (1.4 equiv.), Pd(OAc)_2_ (0.05 equiv.), *rac*-BINAP (1.8 equiv.), toluene, 120 °C, 2 h; (iv) (1) Ba(OH)_2_·8H_2_O (1.5 equiv.), CH_3_OH, 90 °C, 2 h; (2) 1 M aq. HCl; (v) POCl_3_ (34 equiv.), 120 °C, 2.5 h. (vi_b_) Phenol (15 equiv.), Cs_2_CO_3_ (1 equiv.), *N*′,*N′*-diethylpentan-1,4-diamine (4 equiv.), anhydrous DMSO, MW heating (100 W, 100 °C) in a pressurized vial (100 psi), 40 min; (vii) SnCl_2_ (5 equiv.), 37% aq. HCl, 0 °C → 40 °C, 0.5 h; (viii_b_) 17 (1.1 equiv.), NaBH(OAc)_3_ (2 equiv.), 1,2-dichloroethane (DCE), rt, 4 h or 72 h; (ix) hydrazine monohydrate (40 equiv.), THF, 80 °C, 24–48 h; (x) *p*-TSH (1 equiv.), CHCl_3_, rt, 24 h; (xi) NH_2_NH_2_ (10 equiv.), Pd/C (10% wt), CH_3_OH, 80 °C, 1–3 h; (xii_b_) NaBH_4_ (10 equiv.), CH_3_OH, 80 °C, 24 h; (xiii) *N*-(4-bromobutyl)phthalimide (3 equiv.), Et_3_N (3 equiv.), CH_3_CH_2_OH, MW heating (100 W, 120 °C) in a pressurized vial (100 psi), 3 h; (xiv) phthalic anhydride (1 equiv.), 4-aminobutan-1-ol (1 equiv.), dioxane, 100 °C, overnight; (xv) 16 (1 equiv.), Dess–Martin periodinane (DMP, 1.1 equiv.), CH_2_Cl_2_, rt, 24 h.

In face of the above, we decided to work on the optimization of the synthesis route to 6. Therefore, we opted to take advantage of the properties of the nitro group, and replaced 2a and 3a (Table 1, entry 1) by reagents 2c and 3b (Table 1, entry 2). In this case, the nucleophilicity of 2c is not affected by the nitro group that, in turn, makes the bromine in *ortho* position of 3b very electrophilic, which favors the cross-coupling reaction. This led to successful synthesis of 5 in moderate yield (Table 1, entry 2; *η* = 45%) and without the need for steps i, ii and iv shown in Scheme 1. Additionally, the increase of the reaction time from 5 h to 24 h considerably improved the yield (Table 1, entry 3a; *η* = 70%). Despite this clear improvement, the low solubility of 5 makes scale-up challenging, especially concerning its isolation by column chromatography on silica, which reduces the overall yield to below 50% (Table 1, entry 3b). An attempt to overcome this obstacle, by carrying out both the cross-coupling and cyclization steps in a one-pot procedure, avoiding the purification step, was not successful. In view of this, we next tested replacement of the carboxylic acid 2c by the corresponding methyl ester 2b; this allowed production and isolation of 4 in very good overall yield (75%), without any purification issues (Scheme 2, step iii and Table 1, entry 4). Relevantly, the yield was significantly improved by not using any excess of 2b (Table 1, entry 5).

**Table 1:** Optimization of step iii (Scheme 1, part A) in the synthesis route to 6,9-dichloro-2-methoxy-4-nitroacridine 6

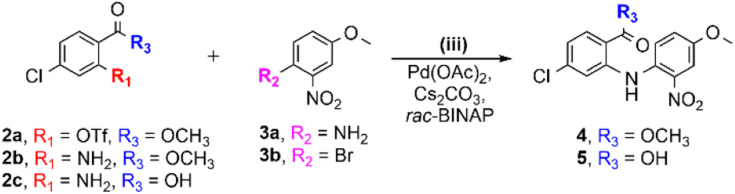					
Entry	Amine (equiv.)	Halogen (equiv.)	Product	Time/h, *T*/°C	Yield/%
1	3a (1.2)	2a (1.0)	4	5, 120	50^b^
2	2c (1.2)	3b (1.0)	5	5, 120	45^a^
3	2c (1.2)	3b (1.0)	5	24, 120	70^a^ (<50)^b^
4	2b (1.2)	3b (1.0)	4	1, 120	75^b^
5	2b (1.0)	3b (1.0)	4	2, 120	90^b^

a Synthesis at the 100 mg scale.

b Synthesis at the 1.5 g scale.

The optimization efforts above targeted production of 6, which bears a chlorine substituent in C-9. Yet, production of the acridine derivative devoid of chlorine in this position is also of great interest for the synthesis of the 4-aminoacridines 15, as it will turn the two reduction steps in Scheme 1 (step x and xii) unnecessary. As such, we addressed the partial reduction of ester 4 into the corresponding aldehyde, which after cyclization using a strong acid like trifluoracetic acid could directly afford compound 13. Therefore, we adopted the methodology reported by Na and co-workers,^19,20^ which makes use of a DIBALH/morpholine system to produce a morpholine amide intermediate that, in the presence of an hydride source, leads to aldehyde formation in mild conditions, short reaction times (<60 min), and almost quantitative yields. Unfortunately, this approach was not successful in our case. Likewise, the reduction of ester 4 to the corresponding alcohol using a sodium borohydride–methanol system, followed by oxidation to the aldehyde,^21–23^ equally failed.

One last optimization effort for the synthesis of 6 regarded the cyclization by dehydrogenation (Scheme 1, step v). Replacement of POCl_3_ by POBr_3_ to produce the Br-substituted acridine in C-9 was attempted, given the fact that bromine is a better leaving group than chlorine which could improve the ensuing aromatic substitutions (Scheme 1, steps vi and x). Unfortunately, these conditions led to a complex mixture of products, amongst which the desired one could not be detected by mass spectrometry (MS) or thin layer chromatography (TLC).

Overall, and regardless of obstacles found, the synthesis route to 6 depicted in Scheme 2 allowed to produce the target compound, 6,9-dichloro-2-methoxy-4-nitroacridine, in only three steps and with an overall yield of 63% (Scheme 2, part B). This is clearly a significant improvement over the previously reported route, where 6 was produced in 5 steps (Scheme 1, part A) with an overall 21% yield.^15,16^

### Optimization of the synthesis route to 4,9-diaminoacridines (10)

The chemical synthesis of 4,9-diaminoacridines 10 had been previously achieved *via* four reaction steps (Scheme 1, steps vi–ix),^15^ starting with a nucleophilic aromatic substitution (S_N_Ar) on 6 using *N*′,*N′*-diethylpentan-1,4-diamine; this was a time-consuming (6 h) procedure comprising activation of 6 by formation of an aryl ether intermediate at C-9, and the subsequent nucleophilic attack by the amine to this intermediate (Scheme 1, step vi). The rate and regioselectivity of this reaction is strongly dependent on acridine substituents, and the presence of the nitro group in the C-4 position of 6 significantly increases its reactivity, which results in the formation of multiple by-products.^10,11^ For this reason, our first optimization attempt consisted in first reducing 6 into 6,9-dichloro-2-methoxy-4-aminoacridine, by use of SnCl_2_, and then perform the S_N_Ar reaction. However, 6,9-dichloro-2-methoxy-4-aminoacridine failed to undergo the desired S_N_Ar, probably due to the electron-donating effect of the amine group in C-4. Therefore, we alternatively focused our optimization efforts on the steps carried out under microwave (MW) heating, as this has very well-known advantages, such as reducing the reaction times, improving yields and purity of the target compounds, and overall simplifying work-up procedures.^24^ As such, we adapted the procedure reported by Staderini and co-workers,^25^ to produce compound 7 using MW microwave heating at 100 °C (Scheme 2, step vi_b_); this reaction showed to be quite time-dependent, since an increase of reaction time from 40 minutes to 1 h about halved the yield from 46% to 24%. Relevantly, conventional heating led to similar yields, but use of MW heating notably reduced the reaction time from 6 h to 40 minutes, and simplified the overall procedure and work-up, by obviating the need for an initial phenol-activation step. We next addressed optimization of the introduction of the aminoalkyl side chain in the C-4 position of the acridine ring of aniline 8; we had previously started by reducing the nitro group in 7 with SnCl_2_/HCl, to produce 8 in 85% yield (Scheme 1, step vii), and next alkylated 8 with *N*-(4-bromobutyl)phthalimide using CH_3_COONa as base (Scheme 1, step viii and Table 2, entry 1). However, this approach required high temperature (120 °C) and excess of the alkylating agent (3 equiv.), which allowed the formation of the di-alkylated product. As such, we started by testing whether reducing the amount of alkylating agent from 3 to 1 molar equivalents could reduce the amount of di-alkylated product and consequently increase the yield of the desired mono-alkylated product, 9. However, although this change did decrease the extension of the side-reaction, it also lowered the yield of formation of 9 from 30% to 10% (Table 2, entry 2). Alternatively, compound 9 was synthesized *via* reductive amination of aldehyde 17, which was first produced by reaction of phthalic anhydride with 4-aminobutan-1-ol (Scheme 2, step xiv; *η* = 70%) followed by selective oxidation with Dess–Martin periodinane (DMP) (Scheme 2, step xv; *η* = 63%).^26^ This method, which uses milder conditions and avoids di-alkylated by-products, successfully afforded 9 in 48% yield, thus offering a significant improvement over our initial approach (Table 2, entry 3 and Scheme 2, step viii_b_). The increase of reaction time and of the amount of aldehyde to 24 h and 3 molar equivalents, respectively, favored formation of by-products with a consequent decrease in the reaction yield to 10% (Table 2, entry 4).

**Table 2:** Optimization of step viii (Scheme 1, part B) in the synthesis route to 4,9-diaminoacridines 10

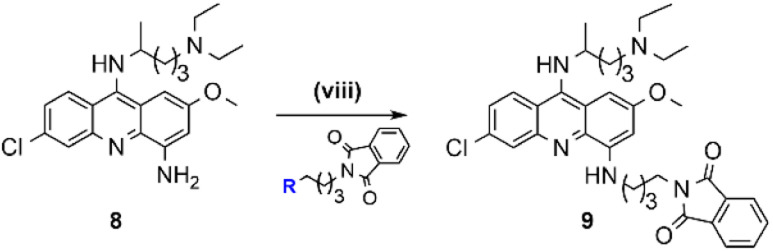					
Entry	Phthalimide	Phthalimide equiv.	Time/h	*T*/°C	Yield/%
1^a^	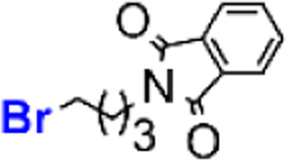	3.0	2.5	120	30
2^a^		1.0	2.5	120	10
3^b^	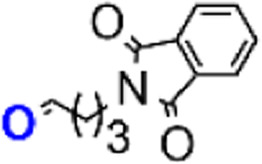	1.1	4.0	rt	48
4^b^		3.0	24	rt	10

a Reaction performed in a MW reactor (100 psi, 100 W) using CH_3_CH_2_OH as solvent.

b Reaction performed at rt using DCE and NaBH(OAc)_3_ as solvent and reducing agent, respectively.

### Optimization of the synthesis route to 4-aminoacridines (15)

Our initial synthesis route to 4-aminoacridines 15 comprised five reactional steps starting from 6, in which reduction of the chlorine and nitro groups in C-9 and C-4, respectively, led to the 4-aminoacridine 12 that was next *N*-alkylated to afford the *N*-phthalimide 14, which was finally converted into 15 by removal of the phthaloyl *N*-protecting group (Scheme 1, steps x–ix).^16^ Our efforts to improve the synthesis of the target 4-aminoacridines 15, which are summarized in Table 3, first focused on the reduction steps. Initial attempts involved the selective remotion of the chlorine in C-9 by reduction with H_2_/Pd–C or with Et_3_SiH/Pd(PPh_3_)_2_Cl_2_; these attempts led to the loss the two chlorines in the acridine systems (Table 3, entry 1 and 2). In view of this, we redirected our focus to the reduction of intermediate 11 that is produced by reacting 6 with *p*-toluenesulfonyl hydrazide (*p*-TSH). The cleavage of the *p*-TSH group and the reduction of the nitro group can be simultaneously achieved by treatment with hydrazine (Table 3, entry 3) or SnCl_2_ (Table 3, entry 4). Alternatively, a two-step procedure can be employed using Na_2_CO_3_ in aqueous ethylene glycol (Table 3, entry 5) followed by reduction with hydrazine (Table 3, entry 7).^16^ As a way of enhancing the yield, we next tested the stepwise reduction of 11 into 13, by use of NaBH_4_ in refluxing CH_3_OH (Scheme 2, step xii_b_ and Table 3, entry 6), which afforded the desired compound in 65% yield, higher than that previously achieved (50%). Encouraged by this result, we next tried once more to improve the conditions for simultaneous reduction of the two groups of interest, *i.e.*, chlorine in C-9 and nitro in C-4. To this end, different combinations of NaBH_4_ with transition-metal salts, such as NiCl_2_·6H_2_O, CuBr_2_, Fe(OTf)_3_, Cu(NO_3_)_2_·3H_2_O, CuSO_4_/CoCl_2_, Ni(OAc)_2_·4H_2_O, Cu(acac)_2_, SnCl_2_·2H_2_O, have been reported in the literature as being capable of reducing nitro groups as well.^27–31^ Therefore, we tested the use of NaBH_4_/Cu(NO_3_)_2_·3H_2_O, NaBH_4_/CuBr_2_ and NaBH_4_/SnCl_2_·2H_2_O combinations, but these failed to promote reduction of the nitro group, thus affording 13 (Table 3, entries 8, 9 and 10). Another three one-pot reactions were attempted whereby other combinations of reducing agents were used, namely, Na_2_CO_3_ in aqueous ethylene glycol followed by hydrazine hydrate (Table 3, entry 11; *η* = 6%); Na_2_CO_3_ in aqueous ethylene glycol followed by SnCl_2_/HCl (Table 3, entry 12; *η* = 13%); and NaBH_4_ followed by hydrazine hydrate (Table 3, entry 13; *η* = 15%). Again, none of these attempts gave better yields than the previous methods for concomitant reduction of both groups, *i.e.*, direct conversion of 11 into 12. As one last attempt to improve production of aminoacridine 12, SnCl_2_ was used to reduce the nitro group (Scheme 2, step xi_b_ and Table 3, entry 14), similarly to what had been carried out in the synthesis route to 4,9-diaminoacridines 10. However, results were not so good in this case, since compound 12 was obtained in only 27% yield; mass spectrometry analysis (data not shown) revealed that reduction of the nitro group into the corresponding aniline was accompanied by the entry of a chlorine atom in C-9. Altogether, this optimization effort enabled us to conclude that the best option to produce 12 is by a two-step procedure where the *p*-TSH group in 11 is first removed by reduction with NaBH_4_ followed by reduction of the nitro group with hydrazine, which allows an improvement of the overall yield from 40% to 52% (Scheme 2, steps xi and xii_b_).

**Table 3:** Optimization of steps x–xii (Scheme 1, part B) in the synthesis route to 4-aminoacridines 15

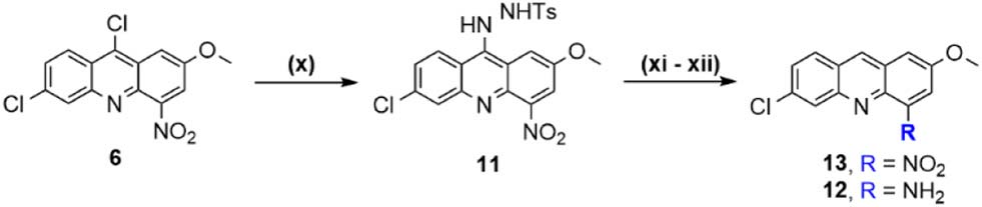							
Entry	Starting reagent	Product	Reducing agent (equiv.)	Catalyst	*T*/°C, time/h	Solvent	Yield/%
1^a^	6	13	H_2_	Pd–C (10 wt%)	rt, 5	CH_3_OH	—
2	6	13	Et_3_Si (1.4)	Pd(PPh_3_)_2_Cl_2_ (1 mol%)	70, 3	CH_3_CN	—
3	11	12	NH_2_NH_2_ (10)	Pd–C (10 wt%)	80, 5	CH_3_OH	30
4	11	12	SnCl_2_ (3.0)	—	40, 0.5	Aq. HCl 37%	18
5	11	13	Na_2_CO_3_	—	95, 1.5	HOCH_2_CH_2_OH/H_2_O (2 : 1)	50
6	11	13	NaBH_4_ (20)	—	80, 24	CH_3_OH	65
7	13	12	NH_2_NH_2_ (10)	Pd–C (10 wt%)	80, 1	CH_3_OH	80
8^b^	11	12	NaBH_4_ (3.0)	Cu(NO_3_)_2_·3H_2_O (15 mol%)	80, 24	CH_3_OH	—
9^c^	11	12	NaBH_4_ (10)	CuBr_2_ (10 mol%)	80, 28	CH_3_OH	—
10^d^	11	12	(1) NaBH_4_ (10)	—	80, 28	CH_3_OH	—
			(2) SnCl_2_·2H_2_O (10)				
11^e^^,^^h^	11	12	(1) Na_2_CO_3_	Pd–C (10 wt%)	(1) 95, 1.5	HOCH_2_CH_2_OH/H_2_O (2 : 1)	6
			(2) NH_2_NH_2_ (10)		(2) 80, 1		
12^f^^,^^h^	11	12	(1) Na_2_CO_3_	—	(1) 95, 1.5	(1) HOCH_2_CH_2_OH/H_2_O (2 : 1)	13
			(2) SnCl_2_ (3.0)		(2) 40, 0.5	(2) HCl	
13^g^^,^^h^	11	12	(1) NaBH_4_ (20)	Pd–C (10 wt%)	80, 25	CH_3_OH	15
			(2) NH_2_NH_2_ (10)				
14	13	12	SnCl_2_ (3)	—	40, 0.5	Aq. HCl 37%	27

a Reaction conditions: H_2_ (50 psi).

b NaBH_4_, Cu(NO_3_)_2_·3H_2_O, CH_3_OH, 80 °C, 24 h.

c NaBH_4_, CuBr_2_, CH_3_OH, 80 °C, 28 h.

d NaBH_4_, CH_3_OH, 80 °C, 24 h, followed by addition of SnCl_2_·2H_2_O, 4 h.

e Na_2_CO_3_ (0.0625 M), HOCH_2_CH_2_OH/H_2_O (2 : 1), 95 °C, 1.5 h, followed by addition of NH_2_NH_2_ and Pd–C, 80 °C, 1 h.

f Na_2_CO_3_ (0.0625 M), HOCH_2_CH_2_OH/H_2_O (2 : 1), 95 °C, 1.5 h, filtration of the black precipitate followed by its dissolution in aqueous HCl 37% → anhydrous SnCl_2_, 40 °C, 0.5 h.

g NaBH_4_, CH_3_OH, 80 °C, 24 h, followed by addition of NH_2_NH_2_ and Pd–C, 80 °C, 1 h.

h One-pot reaction; without isolation of intermediate 13.

Finally, we addressed the improvement of the alkylation of 12 with *N*-(4-bromobutyl)phthalimide to produce 14 (Scheme 1, step xiii). Based on the good results obtained in the optimized synthesis route to 4,9-diaminoacridines 10, we applied reductive amination to obtain 14. However, a significant amount of 12 remained unreacted even after 72 h, probably due to the lowered reactivity of its aniline group, as compared to that in compound 8, its counterpart in the synthesis of 4,9-diaminoacridines 10. This translated into a 30% yield, lower than the 60% achieved *via* bimolecular nucleophilic substitution in the presence of triethylamine (Scheme 1, step xiii). As a very last effort, we still tested the use of acetic acid as catalyst under MW heating,^32^ but these conditions failed to deliver the desired product.

The highest scale attempt for the synthesis of 4-aminoacridine (15) was about 2 g, *i.e.*, starting from approximately 1.8 g of 6,9-dichloroaminoacridine (6). Under these conditions, we were able to obtain 0.5 g of 4-aminoacridine (15). No further scale-up attempts have been performed, but we trust that at least a doubled scale (starting from 4 g) is feasible without significantly affecting the yield.

## Experimental section

### Synthetic procedures§§The procedures described in the Experimental section only refer to the new procedures that led to an improvement on the overall synthesis routes, as depicted in the synthetic route B (Scheme 2).

All chemicals were purchased from Sigma-Aldrich, Abcr, Fluorochem, Fluka, Biochem, Chemopharma, Alfa Aesar, Merck or PanReac AppliChem and used without further purification. The solvents were all of p.a. quality and purchased from VWR International, Carlo Erba, LabChem or Honeywell Riedel-de-Haën. When needed, anhydrous solvents were purchased as such, *i.e.*, not requiring any additional treatment (apart from the use of inert reaction conditions that are identified whenever applicable). Information about the specific brand of each reagent and solvent used is given in detail in the ESI.† Similarly, detailed information about the type of equipments used (such as NMR and mass spectrometers, rotary evaporator, microwave, *etc.*) is included in the ESI.†

#### Route A¶¶The procedures employed in the synthetic route A (Scheme 1) have been already described in detail in ref. 15 and 16.

Compounds 1–15 were prepared following the procedures previously described, and their structural analyses agreed with formerly reported data.^15,16^

#### Route B

##### Synthesis of compound 4 (step iii_b_)

In a 250 mL glass round-bottom flask charged with a magnetic stir bar, 4-bromo-3-nitroanisole (1 equiv., 4.36 mmol, 1.0117 g) was dissolved in toluene (50 mL). Then, Cs_2_CO_3_ (1.4 equiv., 6.10 mmol, 1.9875 g), Pd(OAc)_2_ (0.05 equiv., 0.22 mmol, 0.0494 g) and *rac*-BINAP (0.08 equiv., 0.35 mmol, 0.2179 g) were added. After about 5 min, methyl 2-amino-4-chlorobenzoate (1 equiv., 4.36 mmol, 0.8093 g) was added, and the mixture was heated to 120 °C in an oil bath for 2 h until the starting material disappeared (monitored by TLC). After cooling the mixture to rt, CH_2_Cl_2_ was added (50 mL) and a grey precipitate was collected by gravity filtration and discarded. The filtrate collected in a 250 mL glass round-bottom flask was then evaporated to dryness under reduced pressure and purified by liquid chromatography on a silica gel column using CH_2_Cl_2_/hexane (3 : 1 v/v) as eluent. Orange solid, *η* = 90% (1.3213 g, 3.92 mmol); ^1^H-NMR (400 MHz, DMSO-d_6_) *δ*_H_ 3.85 (3H, s); 3.88 (3H, s); 6.98 (1H, dd, *J* = 8.6 Hz, *J* = 2.0 Hz); 7.20 (1H, d, *J* = 2.0 Hz); 7.36 (1H, dd, *J* = 9.1 Hz, *J* = 3.0 Hz); 7.61 (1H, d, *J* = 3.0 Hz); 7.64 (1H, d, *J* = 9.1 Hz); 7.92 (1H, d, *J* = 8.6 Hz); 10.48 (1H, bs s); ^13^C-NMR (100 MHz, DMSO-d_6_) *δ*_C_ 52.35; 55.97; 109.09; 113.18; 115.17; 119.57; 122.67; 124.32; 128.90; 133.15; 139.01; 140.79; 145.75; 154.56; 166.84; *m*/*z* (ESI-IT MS, +) 337.07 (M + H^+^), M^+^ (C_15_H_13_ClN_2_O_5_) requires 336.05; retention factor (*R*_f_), 0.46 in CH_2_Cl_2_/hexane 3 : 1 (v/v); HPLC-DAD: retention time (RT), 25.620 min, purity degree (peak relative area) 100%.

##### Synthesis of compound 7 (step vi_b_)

A suspension of phenol (15 equiv., 3.81 mmol, 0.3586 g), Cs_2_CO_3_ (1 equiv., 0.25 mmol, 0.0828 g), *N*,*N*-diethylpentane-1,4-diamine (4 equiv., 1.02 mmol, 0.20 mL) and compound 6 (1 equiv., 0.25 mmol, 0.0820 g) in anhydrous DMSO (15 mL) was prepared in a 50 mL glass round-bottom flask. Then, the mixture was transferred to a 35 mL reaction glass vessel, sealed, and submitted to MW heating (100 W) for 40 minutes, at 120 °C. Finished this time, the mixture was cooled to rt, diluted with CH_2_Cl_2_ (25 mL), and, washed with a 2 M aqueous NaOH solution (3 × 50 mL) in a 100 mL glass separatory funnel. The organic layer was collected into a 250 mL Erlenmeyer flask, dried over anhydrous Na_2_SO_4_, filtered by gravity, and evaporated to dryness under reduced pressure to yield the crude product. This was next purified by liquid chromatography on a silica gel column, using CH_2_Cl_2_/CH_3_OH (4 : 1 v/v) as eluent. Red oil, *η* = 48% (51.5 mg, 0.12 mmol); ^1^H-NMR (400 MHz, DMSO-d_6_) *δ*_H_ 0.74 (6H, t, *J* = 7,1 Hz), 1.15–1.36 (2H, m), 1.44 (3H, d, *J* = 6.4 Hz), 1.55–1.64 (1H, m), 1.73–1.82 (1H, m), 2.12–2.17 (2H, m), 2.20 (4H, q, *J* = 7.1 Hz), 3.98 (3H, s), 4.03–4.14 (1H, m), 6.87 (1H, d, *J* = 9.76 Hz), 7.43 (1H, dd, *J* = 9.3 Hz; *J* = 2.2 Hz), 7.85 (1H, d, *J* = 2.2 Hz), 7.86 (1H, d, *J* = 2.6 Hz), 8.05 (1H, d, *J* = 2.6 Hz), 8.32 (1H, d, *J* = 9.32 Hz); ^13^C-NMR (100 MHz, DMSO-d_6_) *δ*_C_ 11.30, 21.85, 23.36, 36.10, 46.07, 51.69, 56.14, 56.41, 104.78, 115.51, 117.34, 118.22, 124.04, 126.42, 127.30, 135.10, 137.10, 148.54, 149.15, 151.62, 152.82; *m*/*z* (ESI-IT MS, +) 445.47 (M + H^+^), M^+^ (C_23_H_30_ClN_4_O_3_) requires 444.19; *R*_f_, 0.45 in CH_2_Cl_2_/CH_3_OH 4 : 1 (v/v); HPLC-DAD: RT, 11.913 min, purity degree (peak relative area) 96%.

##### Synthesis of compound 9 (step viii_b_)

In a 25 mL glass round-bottom flask charged with a magnetic stir bar and put under inert conditions (argon atmosphere), compound 8 (1 equiv., 0.10 mmol, 0.0424 g) was dissolved in DCE (10 mL) and then, compound 17 (1.1 equiv., 0.11 mmol, 0.0239 g) and NaBH(OAc)_3_ (2 equiv., 0.20 mmol, 0.0424 g) were added. After stirring at rt for 4 h, CH_2_Cl_2_ (25 mL) was added and the mixture was washed with a saturated aqueous NaHCO_3_ solution (3 × 25 mL) in a 100 mL glass separatory funnel, dried with anhydrous Na_2_SO_4_, filtered by gravity, and concentrated by evaporation under reduced pressure. Purification by liquid chromatography on a silica gel column using CH_2_Cl_2_/CH_3_OH (4 : 1 v/v) as eluent delivered the target compound; Orange oil, *η* = 48% (30.5 mg, 0.05 mmol); ^1^H-NMR (400 MHz, CDCl_3_) *δ*_H_ 1.19 (6H, t, *J* = 7.6 Hz), 1.32 (3H, d, *J* = 6.3 Hz), 1.60–1.77 (2H, m), 1.80–1.91 (6H, m), 2.85 (2H, t, *J* = 7.5 Hz), 2.93 (4H, q, *J* = 7.1 Hz), 3.31 (2H, t, *J* = 6.1 Hz), 3.76 (2H, t, *J* = 6.6 Hz), 3.93 (3H, s), 3.99–4.08 (1H, m), 6.24 (1H, d, *J* = 2.2 Hz), 6.59 (1H, m), 7.26 (1H, dd), 7.67–7.69 (2H, m), 7.79–7.81 (2H, m), 8.00 (1H, d, *J* = 9.2 Hz), 8.07 (1H, d, *J* = 1.8 Hz); ^13^C-NMR (100 MHz, CDCl_3_) *δ*_C_ 168.53, 158.54, 134.03, 132.17, 125.01, 124.72, 123.29, 55.69, 55.34, 51.77, 46.67, 43.11, 37.80, 35.37, 29.78, 26.55, 26.29, 22.29, 20.94, 8.48, 0.08; *m*/*z* (ESI-IT MS, +) 616.73 (M + H^+^), M^+^ (C_35_H_42_ClN_5_O_3_) requires 615.30; *R*_f_, 0.46 in CH_2_Cl_2_/CH_3_OH 8 : 1 (v/v); HPLC-DAD: RT, 15.347 min, purity degree (peak relative area) 89%.

##### Synthesis of compound 13 (step xii_b_)

A suspension of 11 (1 equiv., 3.75 mmol, 1.7749 g) in CH_3_OH (50 mL) was prepared in a 250 mL glass round-bottom flask charged with a magnetic stir bar, and after that, NaBH_4_ (10 equiv., 37.5 mmol, 1.4186 g) was carefully and slowly added using a spatula during 30 min (paying attention to the exothermic reaction; as during the addition the mixture heats up to boiling). Finished the addition, the mixture was left under stirring until all the starting reagent was consumed (monitored by TLC, approximately 24 h) at 80 °C in an oil bath. After that, the mixture was cooled to rt and CH_3_OH was eliminated by evaporation under reduced pressure. Next, the resulting residue was dissolved in CH_2_Cl_2_ (50 mL) and transferred to a 250 mL glass separatory funnel where it was washed with a saturated aqueous NaHCO_3_ solution (3 × 50 mL). The organic layer was collected in a 250 mL Erlenmeyer flask, dried over anhydrous Na_2_SO_4_, filtered by gravity, and concentrated under reduced pressure. The residue was incorporated in the silica (1 g) and then purified by liquid chromatography on a silica gel column using CH_2_Cl_2_ as eluent. Light-yellow solid, *η* = 64% (0.6914 g, 2.39 mmol); ^1^H-NMR (400 MHz, DMSO-d_6_) *δ*_H_ 4.01 (3H, s), 7.70 (1H, dd, *J* = 9.0 Hz, *J* = 2.1 Hz), 7,80 (1H, d, *J* = 2.7 Hz), 8.16 (1H, d, *J* = 2.0 Hz), 8.24–8.27 (2H, m), 9.18 (1H, s); ^13^C-NMR (100 MHz, DMSO-d_6_) *δ*_C_ 56.49, 108.10, 119.68, 125.46, 127.21, 127.27, 128.07, 130.39, 135.59, 135.82, 136.54, 148.29, 154.76; *m*/*z* (ESI-IT MS, +) 289.53 (M + H^+^), M^+^ (C_14_H_9_ClN_2_O_3_) requires 288.03; *R*_f_, 0.77 in CH_2_Cl_2_; HPLC-DAD: RT, 23.817 min, purity degree (peak relative area) 96.4%.

##### Synthesis of compound 16 (step xiv)

In a 250 mL glass round-bottom flask charged with a magnetic stir bar, 4-aminobutan-1-ol (1 equiv., 6.93 mmol, 0.64 mL) and phthalic anhydride (1 equiv., 6.93 mmol, 1.0270 g) were dissolved in dioxane (100 mL) and left under stirring overnight at 100 °C in an oil bath. Then, dioxane was removed under reduced pressure and 50 mL of CH_2_Cl_2_ was added to the flask. The mixture was transferred to a 250 mL glass separating funnel and washed with a saturated aqueous NaCl solution (3 × 50 mL). The resulting organic phase was collected in a 250 mL Erlenmeyer flask, dried over anhydrous Na_2_SO_4_, filtered by gravity, and evaporated to dryness under reduced pressure. The residue was then purified by liquid chromatography on a silica gel column using CH_2_Cl_2_/CH_3_OH (4 : 1 v/v) as eluent. Yellowish solid, *η* = 70% (1.0633 g, 4.85 mmol); ^1^H-NMR (400 MHz, DMSO-d_6_) *δ*_H_ 1.44–1.37 (2H, m), 1.66–1.58 (2H, m), 3.39 (2H, q, *J* = 2.0 Hz), 3.57 (2H, t, *J* = 7.1 Hz), 4.38 (1H, t, *J* = 5.2 Hz), 7.88–7.81 (4H, m); ^13^C-NMR (100 MHz, DMSO-d_6_) *δ*_C_ 24.71, 29.77, 37.37, 60.15, 122.96, 131.58, 134.34, 167.93; *R*_f_, 0.86 in CH_2_Cl_2_/CH_3_OH 4 : 1 (v/v); HPLC-DAD: RT, 12.280 min, purity degree (peak relative area) 99.8%.

##### Synthesis of compound 17 (step xv)

In a 250 mL glass round-bottom flask charged with a magnetic stir bar and placed under inert conditions (argon atmosphere), compound 16 (1 equiv., 4.51 mmol, 0.9892 g) was dissolved in anhydrous CH_2_Cl_2_ (100 mL) followed by the addition of DMP (1.1 equiv., 4.96 mmol, 2.1037 g). The mixture reacted at rt for 1 h and a white precipitate was formed, which was collected by gravity filtration and discarded. The filtrate was evaporated to dryness under reduced pressure and purified by liquid chromatography on a silica gel column using hexane/AcOEt (1 : 1 v/v) as eluent. Yellowish oil, *η* = 63% (0.6169 g, 2.84 mmol); ^1^H-NMR (400 MHz, DMSO-d_6_) *δ*_H_ 1.84 (2H, p, *J* = 7.0 Hz), 2.54–2.51 (2H, m), 3.59 (2H, t, *J* = 6.8 Hz), 7.88–7.81 (4H, m), 9.64 (1H, t, *J* = 1.1 Hz); ^13^C-NMR (100 MHz, DMSO-d_6_) *δ*_C_ 20.61, 36.77, 122.96, 131.65, 134.30, 167.97, 200.46; *R*_f_, 0.61 in hexane/AcOEt 1 : 1 (v/v).

## Concluding remarks

Acridine-based compounds have for long attracted the interest of researchers across disciplines, and such interest endures due to the unique chemical, physical, and biological properties of those heterocyclic molecules. Our research group makes no exception to this rule, very much driven by our long-term interest in anti-infective agents, including antimalarials inspired in mepacrine.^14–17,33–38^ This led us to advance, in recent years, new 4-aminoacridines (15) and 4,9-diaminoacridines (10) as multi-stage antiplasmodial hits.^15,16^ Although we were successful in addressing their quite challenging synthesis, the overall modest yields obtained as well as harsh and/or time-consuming procedures involved prompted us to work on the optimization of the synthetic routes. The efforts undertaken are herein described and allowed us to make significant progress on several parts of the overall synthesis scheme. As such, the synthesis of compound 6, a common precursor to both families of the target aminoacridines, can now be achieved in three (Scheme 2, part A) instead of five (Scheme 1, part A) steps, using two commercially available reagents and offering an overall yield of 63%, which more than doubles that of the previous approach (24%). Also, the introduction of side chains in the C-9 and C-4 positions of the acridine ring in the route to 4,9-diaminoacridines 10 (Scheme 1, steps vi and viii) could be significantly improved by, respectively, use of MW heating in step vi, and change from a bimolecular nucleophilic substitution into a reductive amination in step viii (Scheme 2, part B, steps vi_b_ and viii_b_); overall, this simplified experimental procedures and considerably reduced reaction time and the need to use high temperatures, while improving yield. Finally, the synthesis route to 4-aminoacridines 15 could also be improved, mainly by a fine tuning of experimental conditions of the reduction steps leading to the 4-aminoacridine intermediate 12 (Scheme 2, part B, steps xi_b_ and xii_b_).

To sum up, after these efforts, 4,9-diaminoacridines 15 can be produced from 6 by four reactional steps (S_N_Ar in C-9, reduction in C-4, reductive amination in C-4, and hydrazinolysis) in 21% overall yield that is about 2-fold higher than that previously reported (13%).^15^ Likewise, 4-aminoacridines 10 can be now delivered from 6 by five reactional steps (S_N_Ar in C-9, reduction in C-9 and C-4, S_N_2 in C-4 and hydrazinolysis) with an overall yield of 34%, also superior to that afforded by the previous synthetic route (21%).^15,16^ Overall, this translates into a more convenient and sustainable production of functionalized acridines, paving the way towards future synthetic approaches targeting acridine-based compounds.

## Conflicts of interest

There are no conflicts to declare.

## Supplementary Material

RA-014-D4RA00091A-s001
