# Supplementary material for: Improved synthesis of antiplasmodial 4-aminoacridines and 4,9-diaminoacridines
Source: RSC Adv. 2024 Feb 19;14(9):6253–61. doi: 10.1039/d4ra00091a (PMC10875606; doi:10.1039/d4ra00091a)
Supplement: RA-014-D4RA00091A-s001 [file RA-014-D4RA00091A-s001.pdf]

Electronic Supplementary Material (ESI) for RSC Advances.
This journal is © The Royal Society of Chemistry 2024

Electronic Supporting Information

Improved synthesis of antiplasmodial 4-aminoacridines and 4,9-diaminoacridines

Mélanie Fonte,*[a] Cátia Teixeira[a,†] and Paula Gomes*[a]

[a] LAQV-REQUIMTE, Departamento de Química e Bioquímica, Faculdade de Ciências, Universidade do Porto, Portugal;

† Current affiliation: Gyros Protein Technologies Inc., Tucson, Arizona, USA.

Contents:

General information 2

Spectral and chromatographic traces for compounds 4, 7, 9, 13, 16, 17 (Figures S1-S21) 3

General information.

All solvents and commercial chemicals were purchased from Sigma-Aldrich (phenol, $Pd(OAc)_2$, *N,N*-diethylpentan-1,4-diamine, $NH_2NH_2.2H_2O$, Pd-C, Cs_2CO_3, 2-amino-4-chlorobenzoic acid, phthalic anhydride, $SnCl_2.2H_2O$, $Pd(PPh_3)_2Cl_2$, 4-bromo-3-nitroanisole, $Cu(NO_3)_2.3H_2O$, $CuBr_2$, and $NaBH(OAc)_3$), Fluorochem (Dess-Martin periodiane and *N*-(4-bromobutyl)-phthalimide), Fluka (4-aminobutanol, $NaBH_4$, Et_3SiH, and anhydrous $SnCl_2$), Biochem Chemopharma ($NaHCO_3$), Abcr (*rac*-BINAP), VWR international (HCl 37 %; HPLC gradient grade, LC-MS grade, and p.a. quality solvents), Merck (CH_3COONa), Fisher Chemical (glacial acetic acid), Alfa Aesar (methyl 2-amino-4-chlorobenzoate), PanReac AppliChem (Na_2SO_4 and NH_3 25 %), Carlo Erba (toluene), LabChem (CH_3OH, CH_2Cl_2, hexane, AcOEt, diethyl ether), Honeywell Riedel-de-Haën (dioxane and dichloroethane), and CortecNet (deuterated solvents).

Aluminium foils coated with silica-gel 60 F254 from Merck were used for TLC analysis (retention factor, R_f). TLC chromatograms obtained were revealed with ultra-violet light (254 nm), using a Viber Lourmat lamp, model CN-6 and/or using a phosphomolybdic acid stain (15 mL of phosphomolybdic acid solution (20% wt in ethanol) and 85 mL of ethanol).

The purification of the compounds was carried out via normal phase liquid chromatography in glass columns charged with silica gel 60, from Sigma Aldrich, as solid phase. A mixture of solvents (identified in each case) was used as the mobile phase (eluent).

The synthesis of compound **7** was carried out in a CEM microwave model Discovery S.

Electrospray ionization-ion trap mass spectrometry (ESI-IT MS) analyses (direct injection with detection in positive mode) were performed with solutions of the pure products in p.a. methanol, using a Finnigan Surveyor LCQ DECA XP MAX spectrometer, at the Department of Chemistry and Biochemistry of the Faculty of Sciences of the University of Porto.

The determination of the retention time (RT) and purity degree of the compounds was carried out by analytical reverse-phase high-performance liquid chromatography (RP-HPLC), using a Hitachi-Merck Elite LaChrom system equipped with an L-2130 quaternary pump, an L-2200 thermostatted (Peltier effect) automated sampler, and an L-2455 diode-array detector (DAD). Samples were prepared by dissolution of compounds in acetonitrile and injected in an end-capped octadecylsilane (C18) column Purospher star RP-C18 of 125 × 4.0 mm and 5 µm pore size. A gradient elution (1 to 100 % of acetonitrile in 0.05% aqueous trifluoroacetic acid) was carried out for 30 minutes, at a flow rate of 1 mL/min, and detection was set at 290 nm.

^{1}H-NMR and ^{13}C-NMR spectra were acquired on a Bruker Avance III spectrometer at frequencies of 400 mHz and 100 mHz, respectively, and samples were prepared in either DMSO-d_6 or $CDCl_3$, using tetramethylsilane (TMS) as an internal reference. Chemical shifts for protons and carbon are reported in parts per million (ppm) downfield and are referenced to residual protium in the NMR solvent. Data are represented as follows: chemical shift, integration, multiplicity (br = broad, s = singlet, d = double, t = triplet, q = quartet, m = multiplet), coupling constants in Hertz (Hz).

Methyl 4-chloro-2-[(4-methoxy-2-nitrophenyl)amino]benzoate (4)

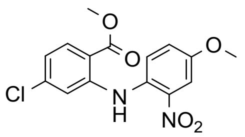

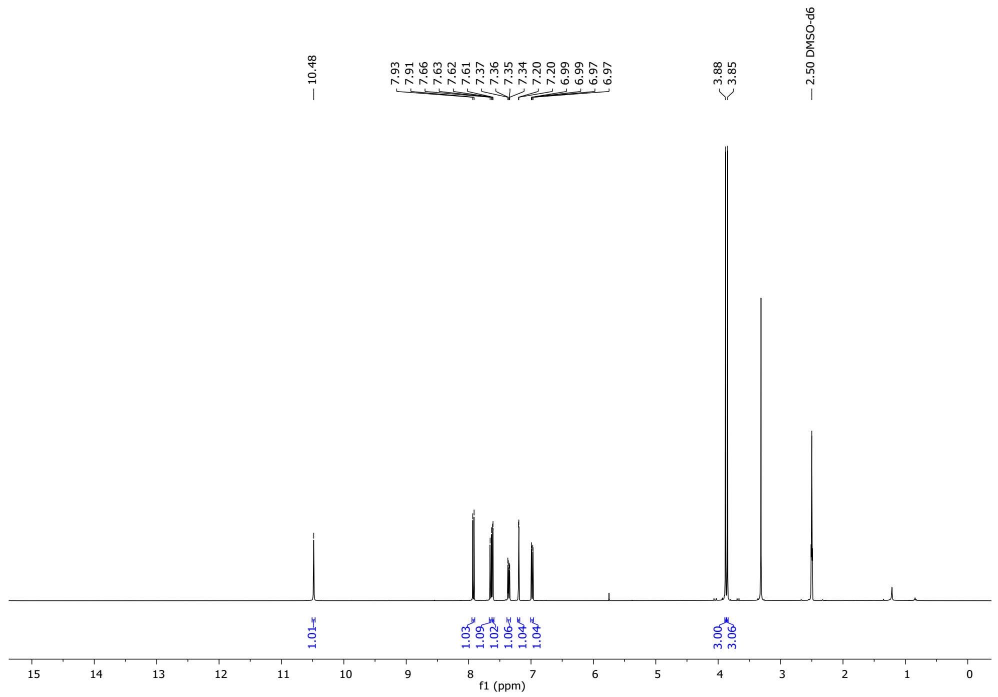

Figure S1. ^{1}H-NMR spectrum (400 MHz, DMSO-d$_6$) of **4**.

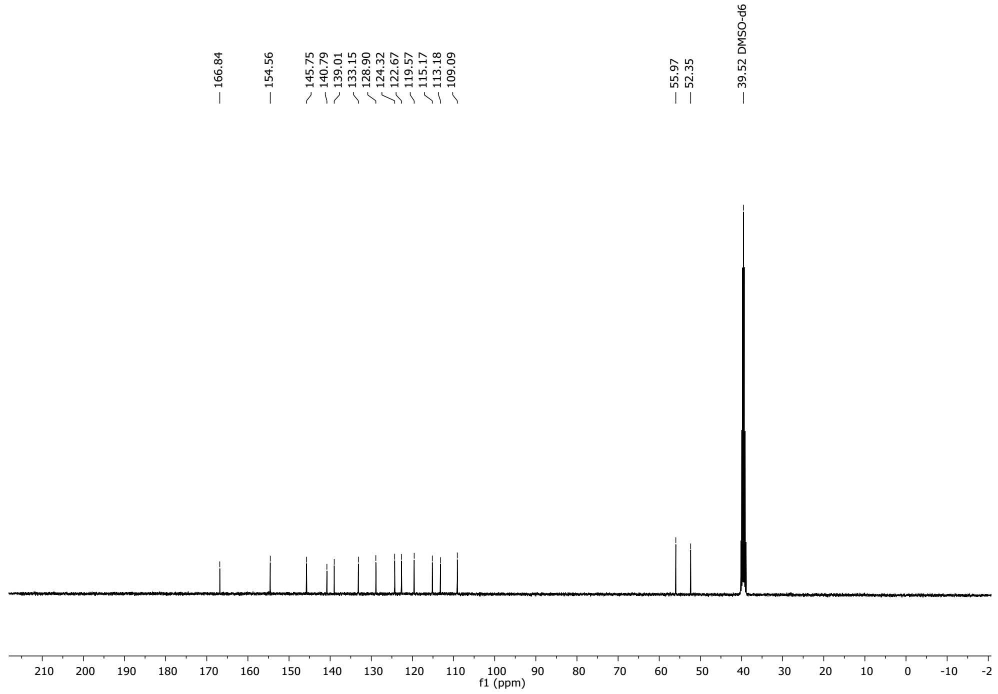

Figure S2. ^{13}C- NMR spectrum (100 MHz, DMSO-d$_6$) of **4**.

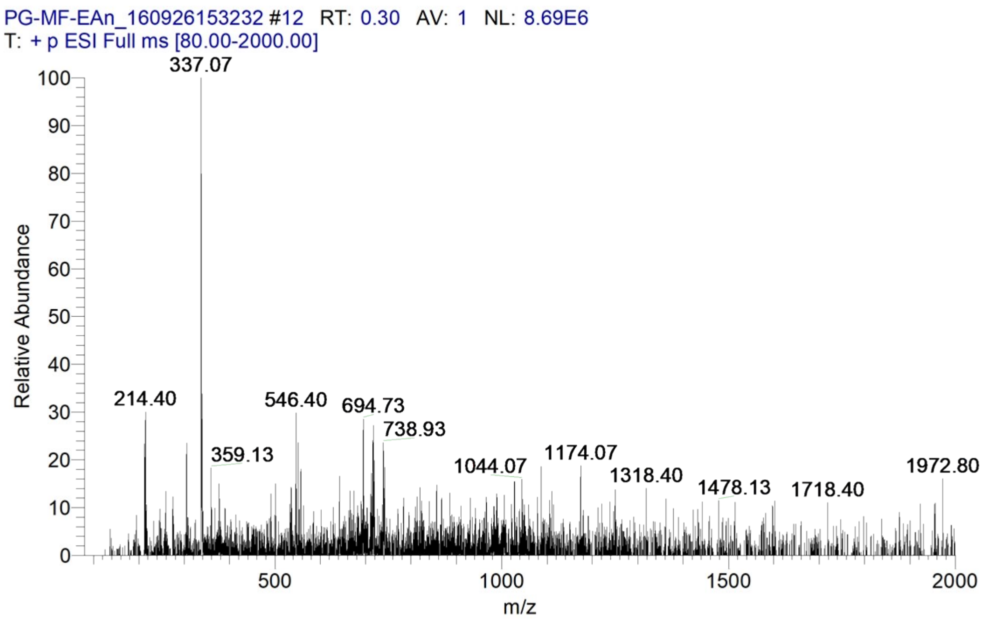

Figure S3. ESI-IT (+) mass spectrum of **4**.

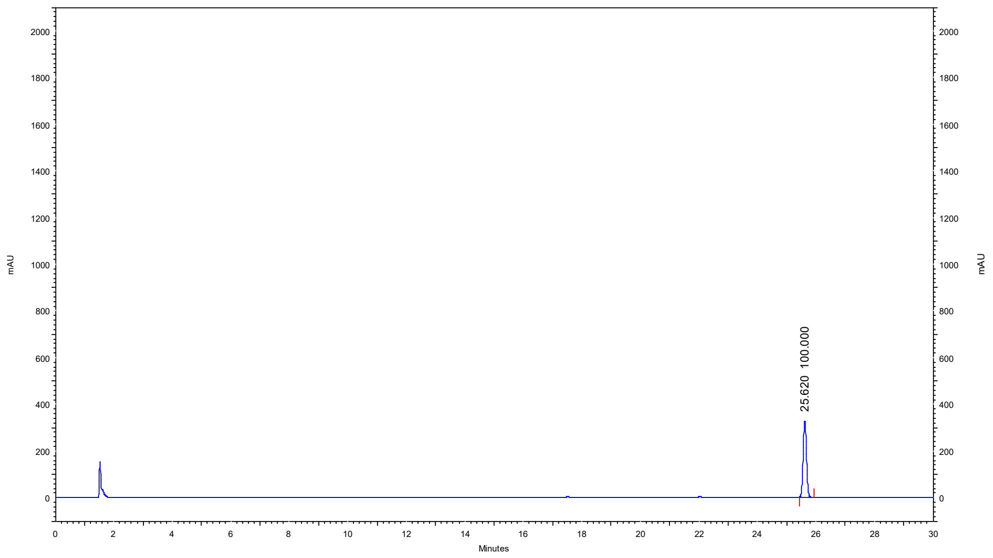

Figure S4. HPLC trace of **4**.

6-chloro-9-[*N*-(4-(diethylamino)pent-2-yl]amino-2-methoxy-4-nitroacridine (7)

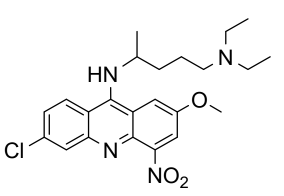

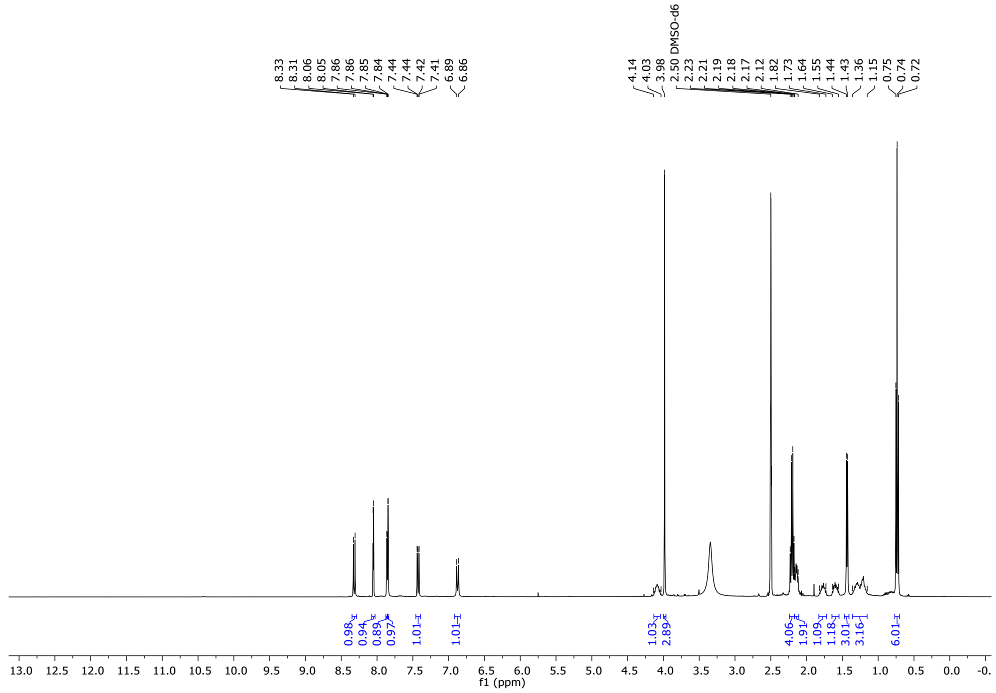

Figure S5. ^{1}H-NMR spectrum (400 MHz, DMSO-d$_6$) of **7**.

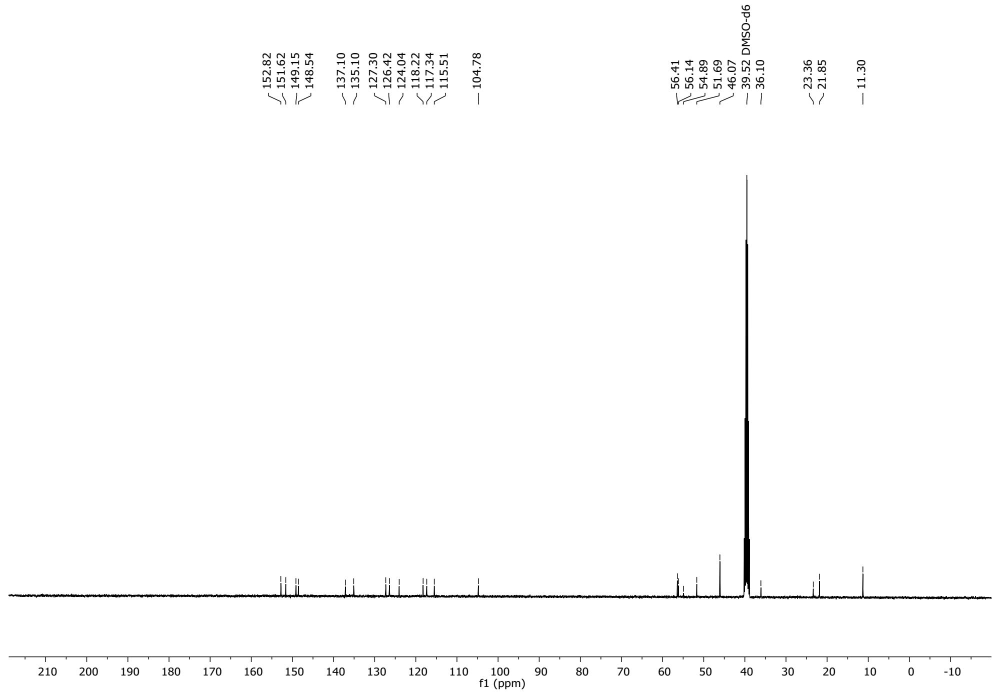

Figure S6. ^{13}C-NMR spectrum (100 MHz, DMSO-d$_6$) of **7**.

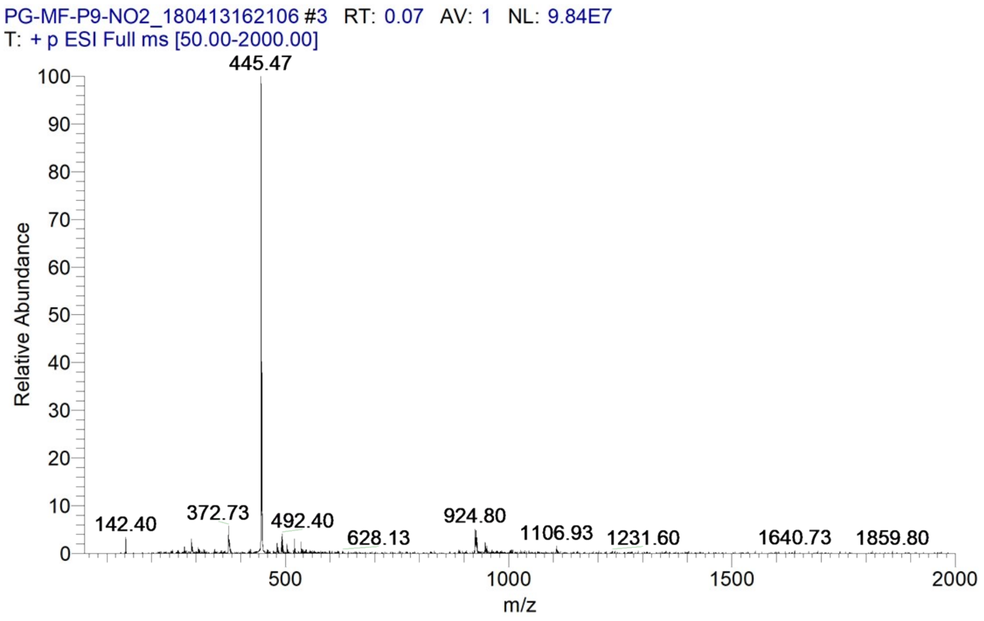

Figure S7. ESI-IT (+) mass spectrum of **7**.

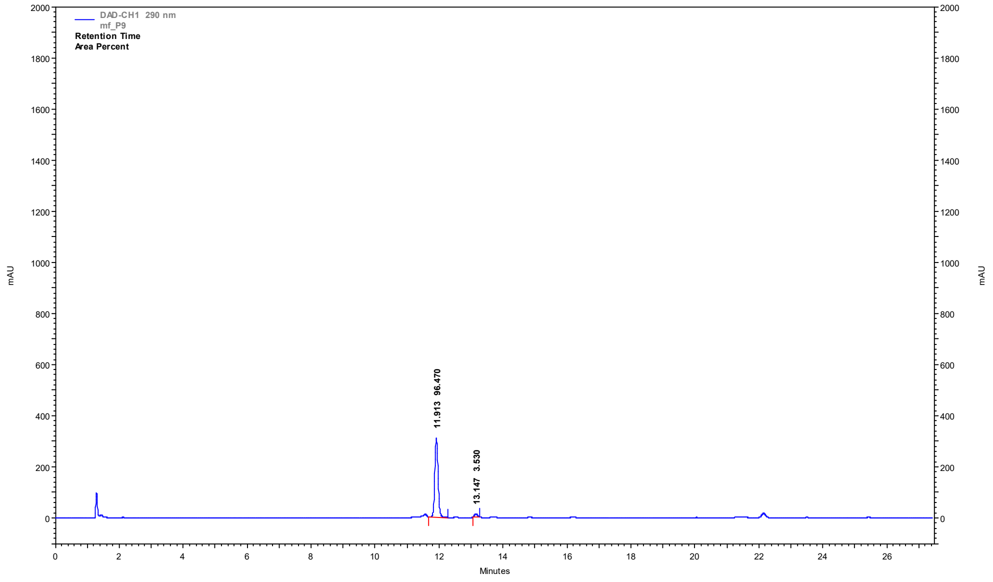

Figure S8. HPLC trace of **7**.

6-chloro-9-[*N*-(4-(diethylamino)pent-2-yl]amino-4-(4-phthalimidobutyl)amino-2-methoxy-acridine (9)

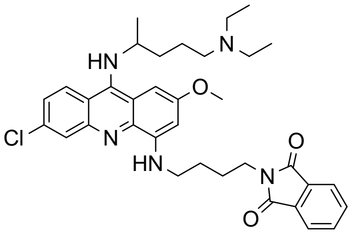

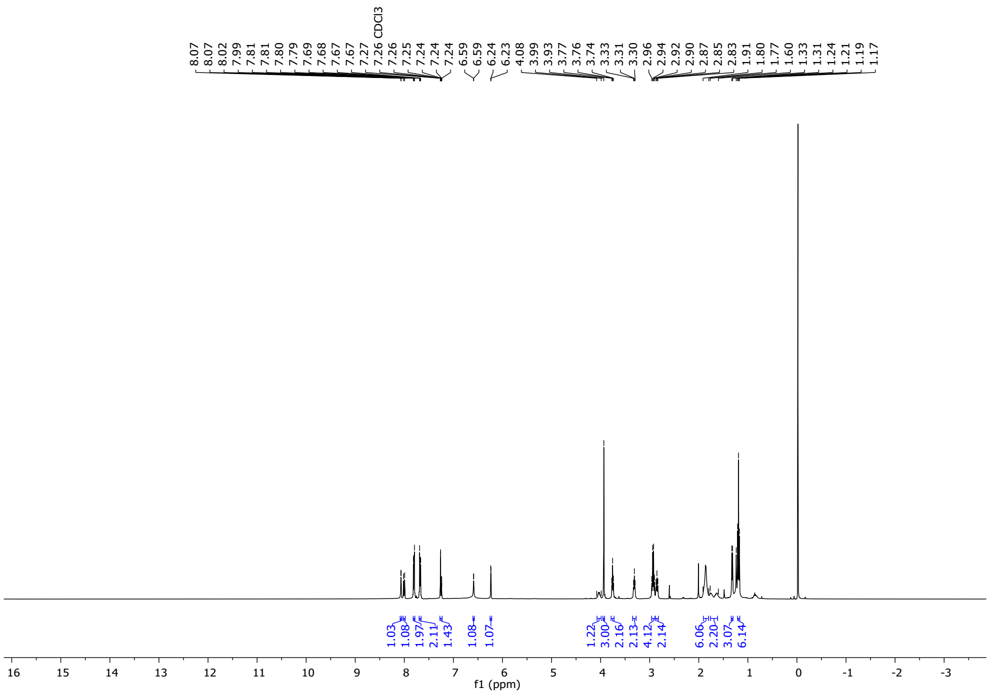

Figure S9. ^{1}H-NMR spectrum (400 MHz, $CDCl_3$) of **9**.

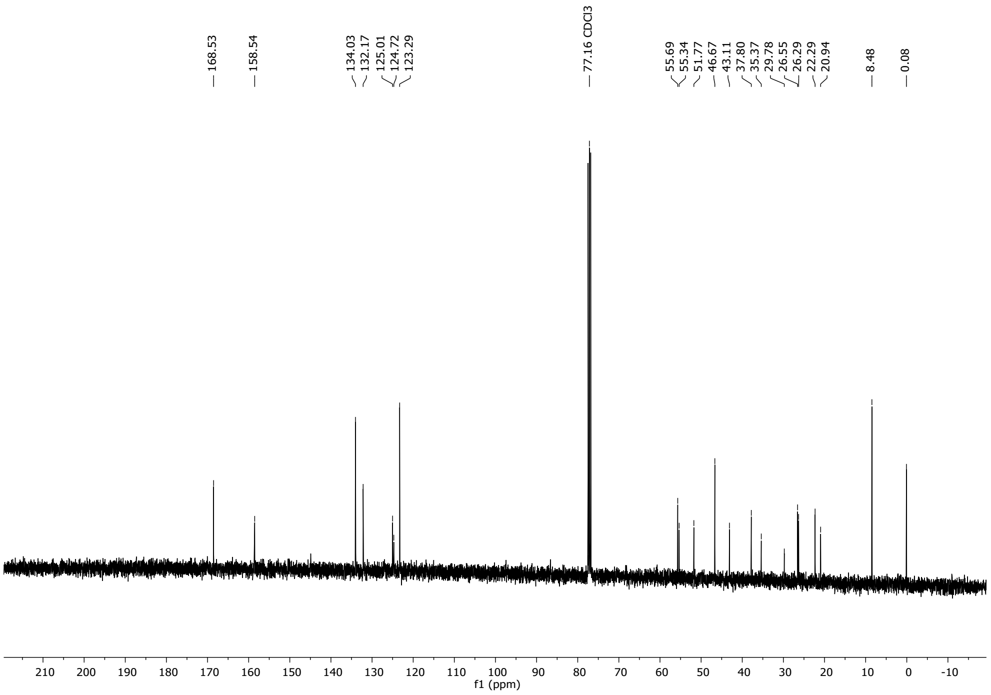

Figure S10. ^{13}C-NMR spectrum (100 MHz, $CDCl_3$) of **9**.

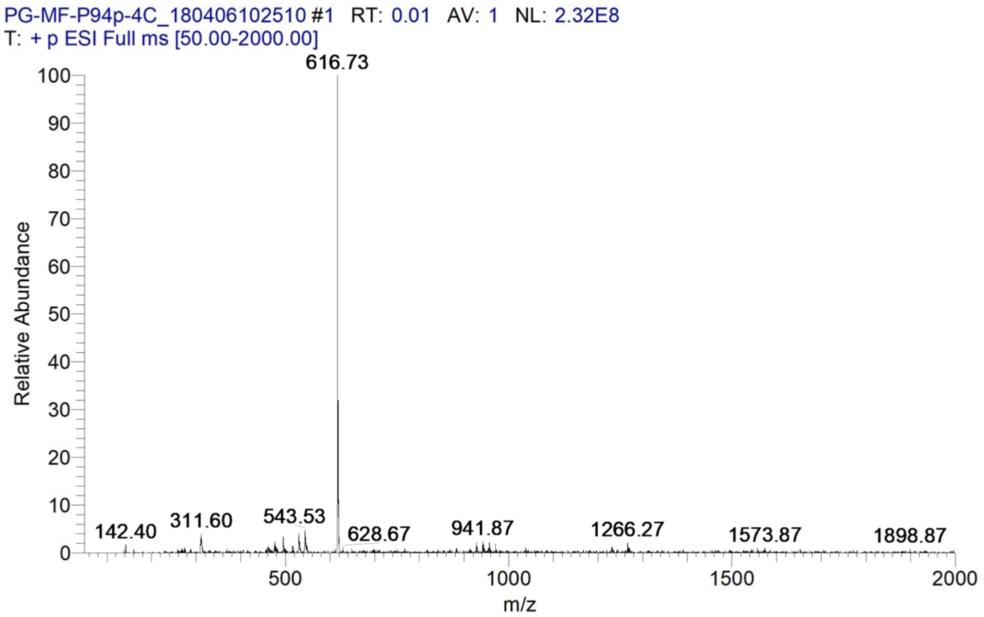

Figure S11. ESI-IT (+) mass spectrum of **9**.

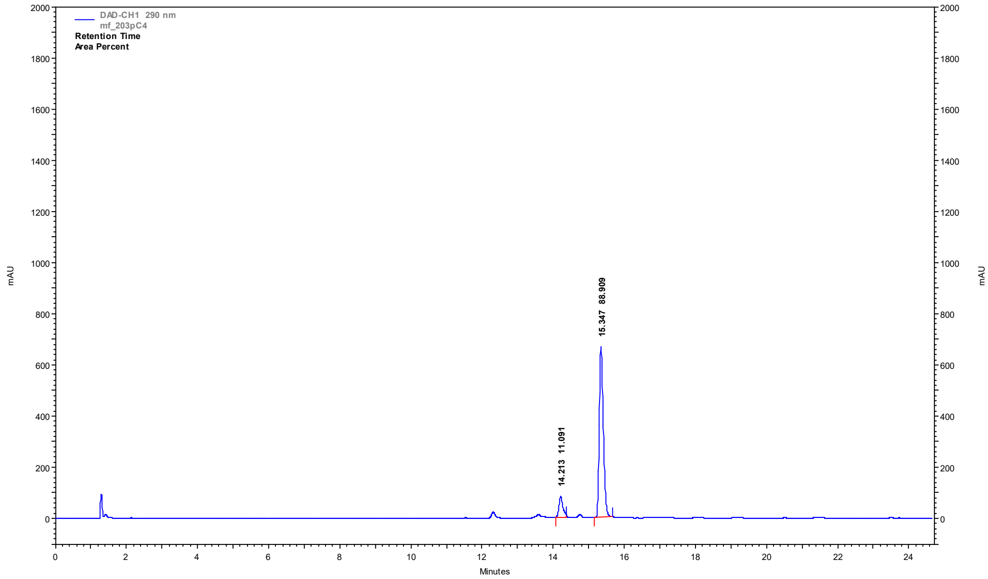

Figure S12. HPLC trace of **9**.

6-chloro-2-methoxy-4-nitroacridine (13)

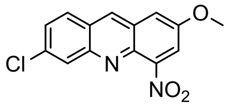

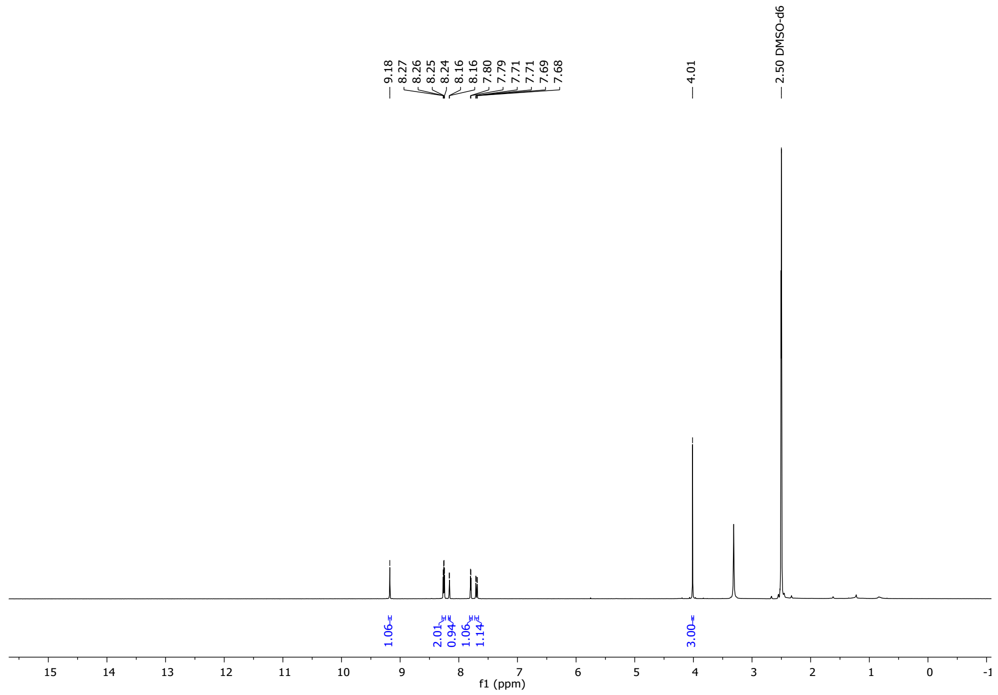

Figure S13. ^{1}H-NMR spectrum (400 MHz, DMSO-d$_6$) of **13**.

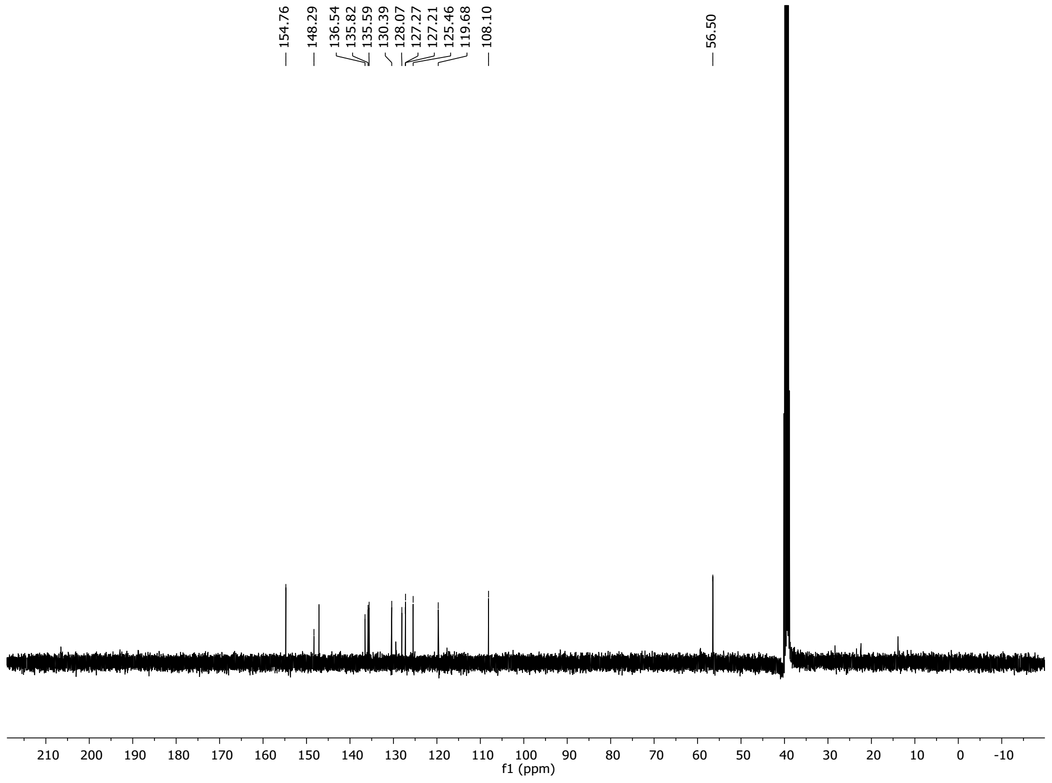

Figure S14. ^{13}C-NMR spectrum (100 MHz, DMSO-d$_6$) of **13**.

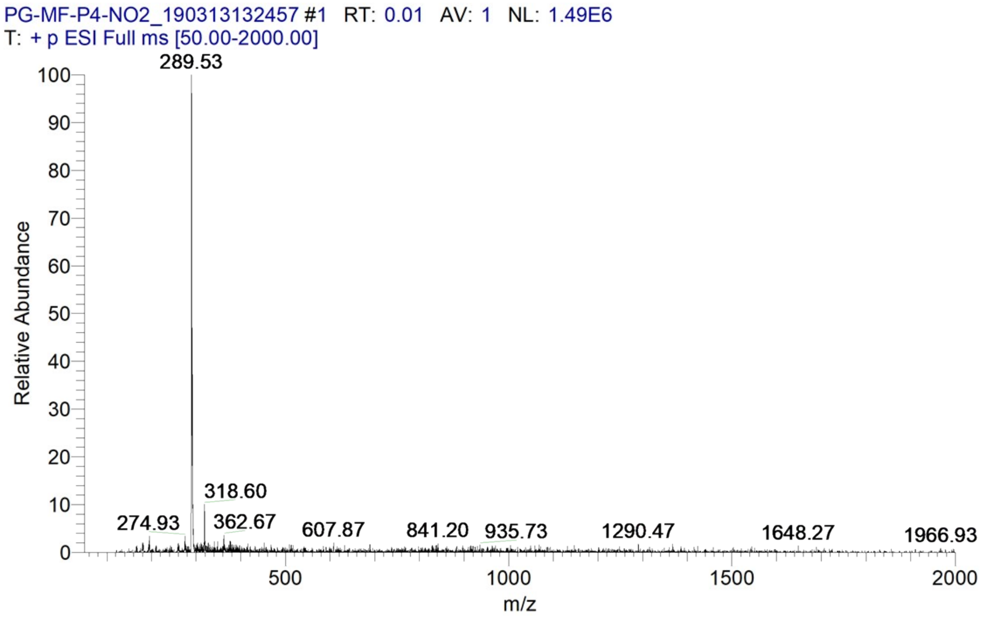

Figure S15. ESI-IT (+) mass spectrum of **13**.

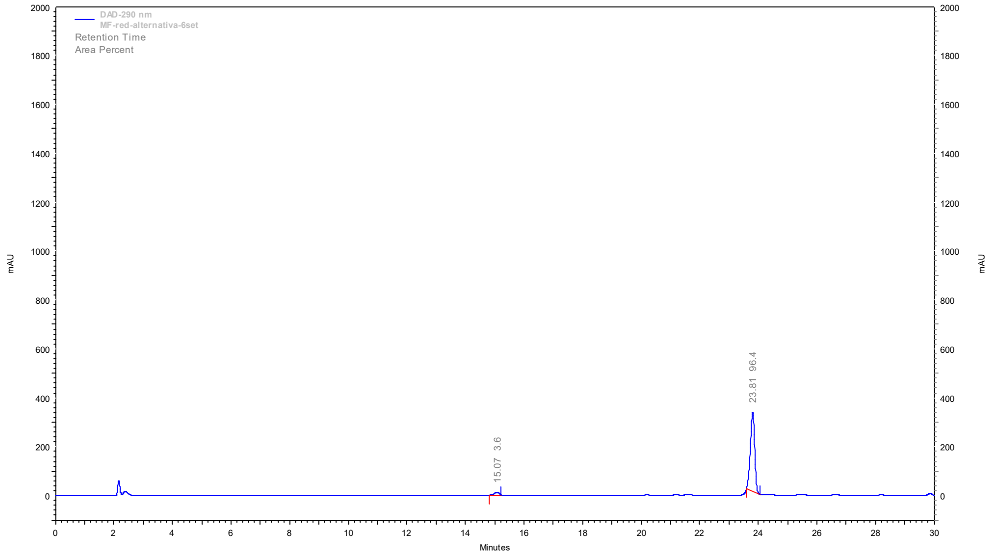

Figure S16. HPLC trace of **13**.

N-(4-hydroxybutyl)phthalimide (16)

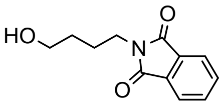

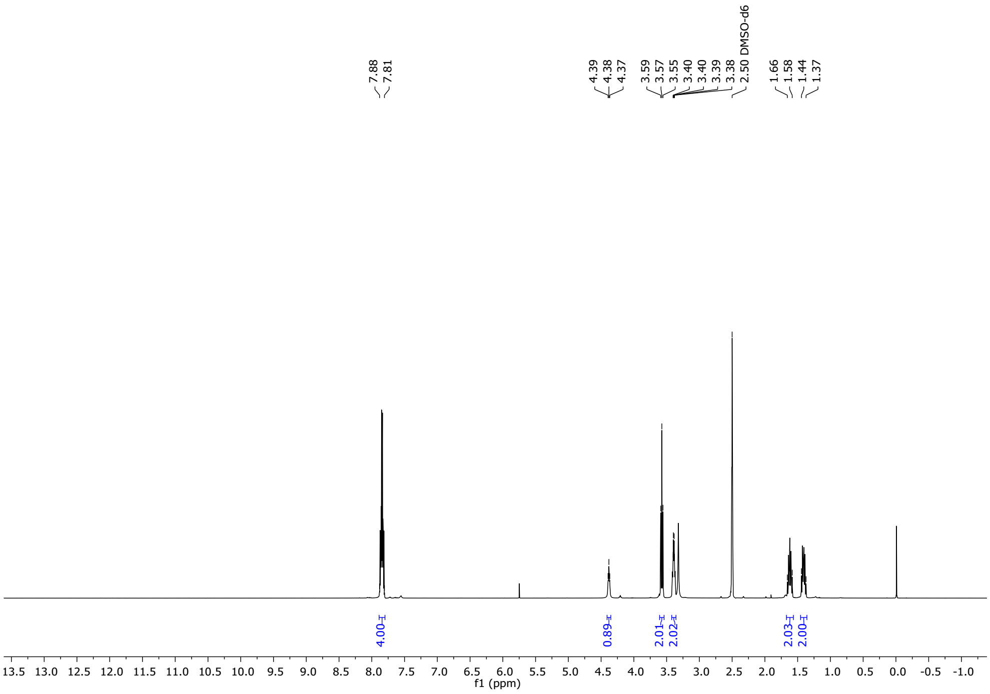

Figure S17. ^{1}H-NMR spectrum (400 MHz, DMSO-d$_6$) of **16**.

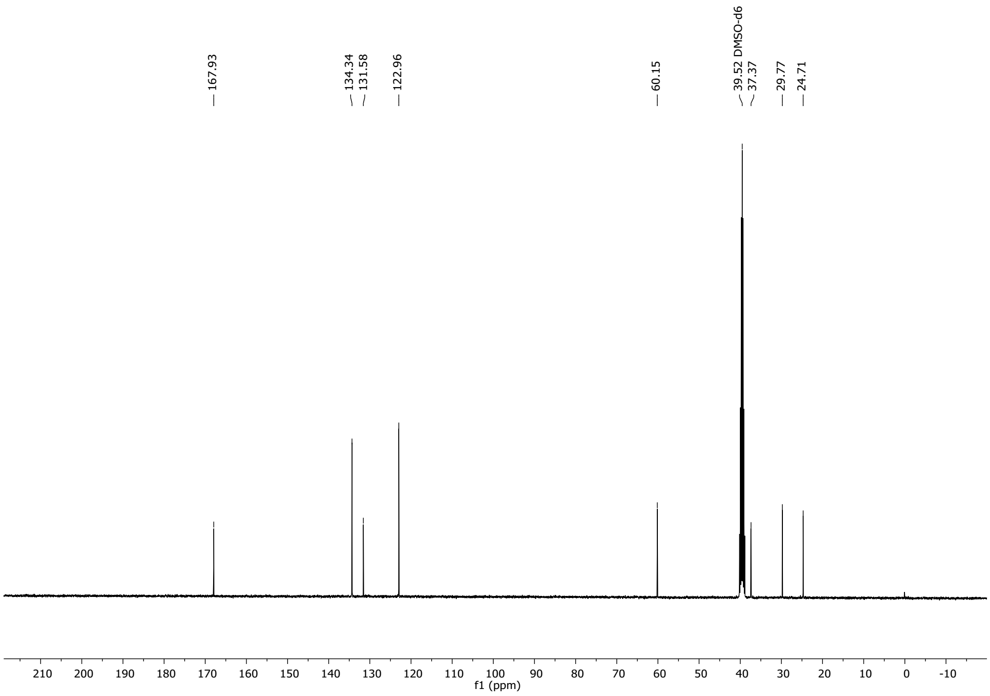

Figure S18. ^{13}C- NMR spectrum (100 MHz, DMSO-d$_6$) of **16**.

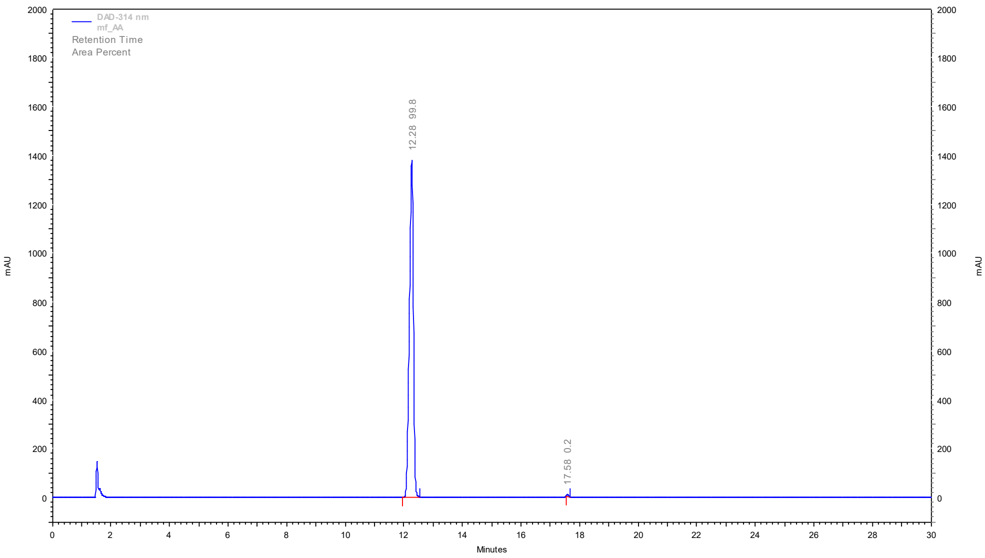

Figure S19. HPLC trace of **16**.

Y-Phthalimidobutyraldehyde (17)

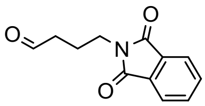

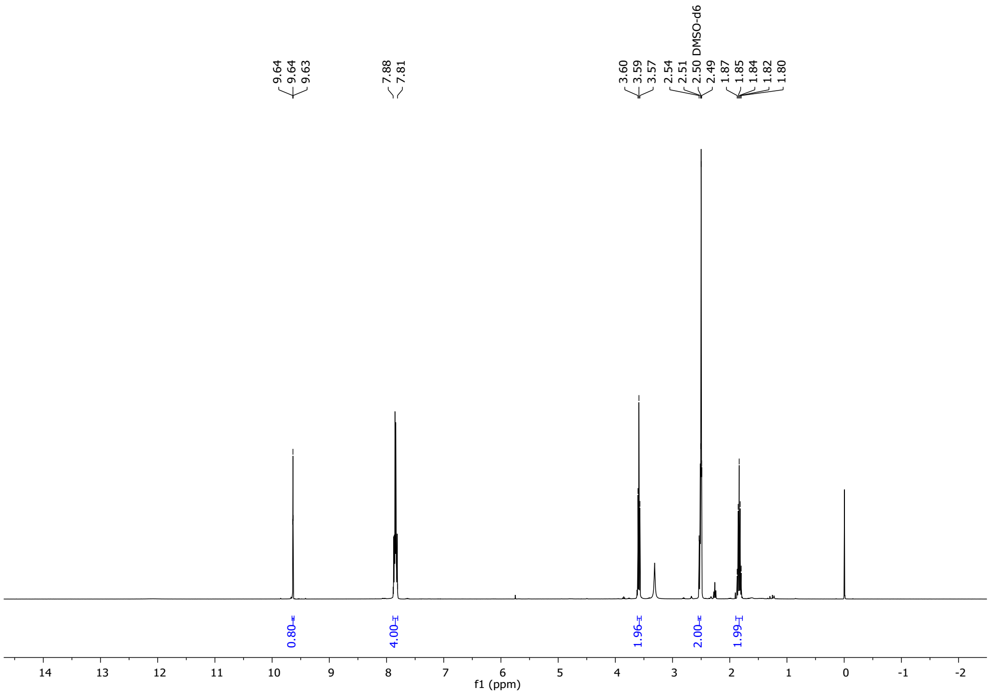

Figure S20. ^{1}H-NMR spectrum (400 MHz, DMSO-d$_6$) of **17**.

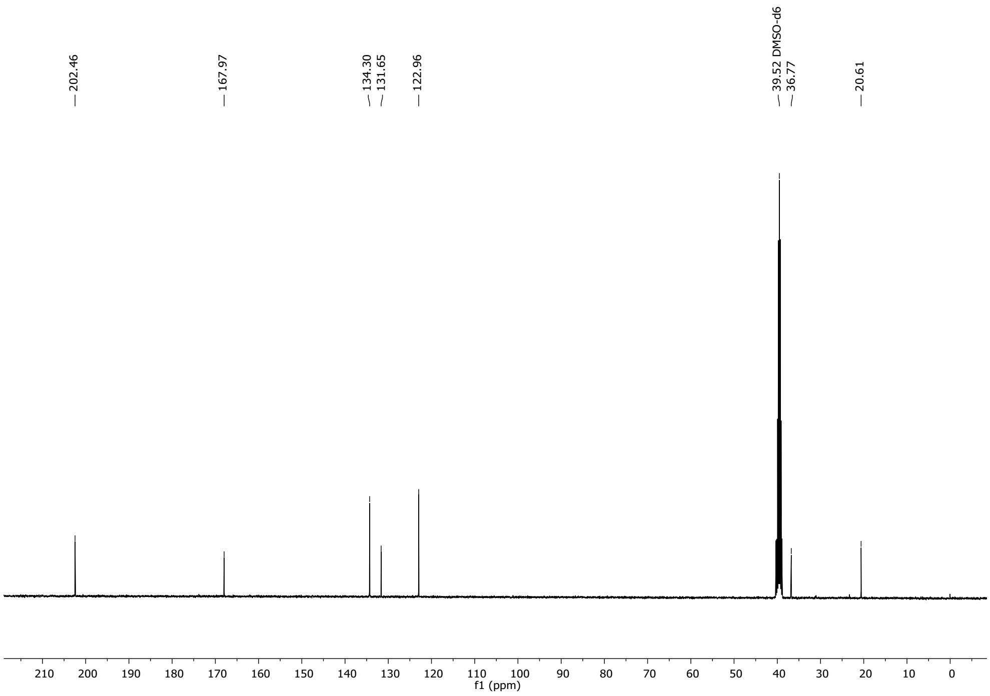

Figure S21. ^{13}C-NMR spectrum (100 MHz, DMSO-d$_6$) of **17**.
